# Mechanotransduction Circuits in Human Pathobiology

**DOI:** 10.3390/ijms25073816

**Published:** 2024-03-29

**Authors:** Antonios N. Gargalionis, Kostas A. Papavassiliou, Athanasios G. Papavassiliou

**Affiliations:** 1 Department of Biological Chemistry, Medical School, National and Kapodistrian University of Athens, 11527 Athens, Greece; 2 ‘Sotiria’ Hospital, Medical School, First University Department of Respiratory Medicine, National and Kapodistrian University of Athens, 11527 Athens, Greece; konpapav@med.uoa.gr

It is widely acknowledged that mechanical forces exerted throughout the human body are critical for cellular and tissue homeostasis. Even during organ development inside the uterus, the application of balanced physical stimuli is required for normal tissue differentiation in the course of embryogenesis. Furthermore, tight control of physical forces and mechanical cues is also crucial for the survival of cells, tissues, and organs during the adult life [1,2,3]. To this end, tissues depend on the degree of adaptability of their cells to the external dynamic mechanical environment. Cells are subjected to and sense certain mechanical forces which modulate their functions. Typical forces applied on cells include blood, lymph, and interstitial fluid shear stress which originates from the fluid flow in the respective circulatory and lymphatic systems, as well as in the interstitial space [4,5]. Cells are further exposed to hydrostatic pressure from fluid that accumulates in the interstitial space and in organs like the urinary bladder [6,7,8,9]. They are able to experience tensile forces, which are also known as mechanical stretch, and compressive forces, which are predominantly observed when there is large-scale tissue deformation during contraction of muscles, movement of joints, cardiovascular remodeling, compressive burden on cartilage and bone, as well as shear stress [10,11]. Other types of forces include mechanical stimuli from cell-to-cell interactions within the respective cell microenvironment and mechanical forces applied when cells are migrating through spaces with physical confinement [12].

Mechanotransduction is the process through which cells convert these mechanical forces to signal transduction mechanisms, thereby triggering a cellular response. To achieve this, cells have developed evolutionary conserved mechanosensitive tools to mediate mechanotransduction [13]. Mechanosensitive ion channels are critical effectors of mechanosignaling and they are mostly associated with regulation of intracellular concentration of calcium ions, as it has been documented for Piezo and transient receptor potential (TRP) families of ion channels [1,13,14]. Mechanosignaling-associated elements include mechanosensitive receptors and membrane-bound proteins like integrins, G protein-coupled receptors (GPCRs), cadherins, focal adhesion kinase (FAK), and Src kinases [2]. Primary cilia is an additional structure of the apical membranes in many types of epithelial cells, through which they are able to sense and transmit mechanical signals to elicit a cellular effect [15]. Mechanotransduction ultimately integrates to mechano-induced transcription factors/cofactors, such as yes-associated protein (YAP) and transcriptional coactivator with PDZ-binding motif (TAZ) (end effectors of the Hippo signal transduction cascade that function through the TEA domain family members (TEAD) transcription factors), hypoxia-inducible factor-1α (HIF-1α), and nuclear factor kappa-light-chain-enhancer of activated B cells (NF-κB) [16,17]. Mechanistic studies reveal specific signaling pathways which are involved in mechanotransduction and related pathobiologies. These pathways encompass the Hippo, RhoA/Rho-associated protein kinase (ROCK), transforming growth factor-*β* (TGF-*β*)/Smad, Janus kinase (JAK)/signal transducers and activators of transcription (STAT), Wnt/*β*-catenin, and mitogen-activated protein kinase (MAPK) signaling cascades [1,18,19,20]. Aberrant regulation of these pathways has been associated with the pathophysiology of a broad spectrum of diseases. Mechanobiology-related disorders emerge usually in tissues with well-defined mechanical properties, such as in the heart, bone, and cartilage. Distorted mechanosignaling has been implicated in congenital heart disease, pathological heart hypertrophy, atherosclerosis, liver, renal, pulmonary, cardiac injury and fibrosis, osteoarthritis, polycystic kidney disease, and cancer [1,2,3].

Within this group of disorders, the role of extracellular matrix (ECM) is prominently linked to dysregulation of mechanotransduction. Cellular dynamics face robust alterations in bone cells, endothelial cells, cancer cells, and cells from their microenvironment. Forces applied to and generated from the ECM have been highlighted as a key regulator of cell mechanobiology. Underpinning mechanisms are thoroughly investigated especially during tumorigenesis. Changes in ECM stiffness and composition as the tumor expands are perceived by integrins and other mechanosensitive membrane receptors to modulate cancer and stromal cell properties [1,2,3,11]. Tissue-dependent increase or reduction in ECM rigidity fosters cancer cell proliferation, vascularization, invasion, and metastasis [21]. Mounting evidence suggests that ECM stiffness is implicated in various aspects of solid tumor pathology, as well as in treatment responses to current therapeutic regimens. For instance, ECM regulates cell proliferation and expression of programmed death-ligand 1 (PD-L1) through YAP upregulation in lung cancer cells [22] and is associated with nasopharyngeal carcinoma (NSC) aggressive cellular features through upregulation of cortactin and polypyrimidine tract binding protein 2 (PTBP2) [23]. On the other hand, hepatocellular carcinoma (HCC) cells and HCC cancer stem cells present high motility, invasion, and metastatic potential with reduced stiffness, a process mediated by activation of c-Jun N-terminal kinase (JNK) [24]. Another cancer hallmark, the function of *RAS* oncogenes and concomitant MAPK pathway upregulation, has been linked to changes in actomyosin contractility, thus altering the sensing ability of stiffness. As recently demonstrated, inhibition of *KRAS^G12D^* targets integrin subunit beta 1 (ITGB1; also known as CD29) and suppresses tumor growth in vitro and in vivo through regulation of YAP and TAZ in pancreatic ductal adenocarcinoma (PDAC) [25,26]. Moreover, an emerging feature of mechanosensitivity is extracellular viscosity, which seems to interact with ECM stiffness and potentiate mechanotransduction. Specifically, in the pre-cirrhotic liver there is augmented ECM viscoelasticity from the accumulation of advanced glycation end products (AGEs), which promotes HCC through the integrin-*β*1-tensin-1-YAP pathway [27].

Current therapeutic approaches exploit features of mechanοtransduction to selectively administer treatments and target molecules of the mechanosignaling machinery. A representative study demonstrates that mechanical cyclic stretching induces apoptosis of tumor cells through modulation of calcium influx by Piezo1 [11]. Further experimental evidence reveals that mechanoresponsive stem cells can be reprogrammed to regenerate bone via FAK signaling and can also sense tissue stiffness to deliver therapeutics directly to cancer metastases in vivo [28,29]. Various ongoing clinical trials exist to assess the impact of hindering mechanosensitive molecules on the progression of diseases. Compounds targeting integrins are evaluated in solid tumors, inflammatory bowel diseases, and pulmonary fibrosis. Drugs targeting YAP/TAZ and TEAD transcription factors are also tested in advanced solid tumors [1]. Finally, an ever-growing volume of data implies that mechanotransduction in the tumor microenvironment is engaged in mechanisms of resistance to chemotherapy and immunotherapy. Components of mechanosignaling are bound to the anti-cancer immunity mechanisms and facilitate immunotherapy resistance, mainly via alterations of ECM stiffness [30]. Therefore, mechanotransduction offers potential routes to overcome drug resistance through combinatorial treatments.

In the future, the clinical management of several diseases will incorporate mechanotherapeutics that exploit the physical associations of cells with their microenvironment.
